# Supplementary material for: Systematic over-expression of secondary metabolism transcription factors to reveal the pharmaceutical potential of Aspergillus nidulans
Source: Commun Biol. 2025 Oct 9;8:1444. doi: 10.1038/s42003-025-08840-z (PMC12511407; doi:10.1038/s42003-025-08840-z)
Supplement: Supplementary file 2 — Supplementary Information [file 42003_2025_8840_MOESM2_ESM.pdf]

Systematic over-expression of secondary metabolism transcription factors to reveal the pharmaceutical potential of *Aspergillus nidulans*

Shuhui Guo[a,g,*], Lakhansing Pardeshi[b], Longguang Qin[a], Chris Y. Cheung[c], Xiaofeng Liu[a], Lu Fan[a], Chi Cheng Mok[a], Chirag Parsania[a], Zhiqiang Dong[a], Ben C.B. Ko[c,d], Kaeling Tan[a,b], and Koon Ho Wong[a,e,f,*]

[a]Faculty of Health Sciences, University of Macau, Macau SAR, China.
[b]School of Health and Nursing, Wuxi Taihu University, Wuxi, China.
[c]Genomics, Bioinformatics & Single Cell Analysis Core, Faculty of Health Sciences, University of Macau, Macau SAR, China.
[d]Department of Applied Biology and Chemical Technology, The Hong Kong Polytechnic University, Hong Kong SAR, China.
[e]Institute of Translational Medicine, Faculty of Health Sciences, University of Macau, Macau SAR, China.
[f]MoE Frontiers Science Center for Precision Oncology, University of Macau, Macau SAR, China.

[*]For correspondence, please contact koonhowong@um.edu.mo and guosh@wxu.edu.cn.

Supplementary figures

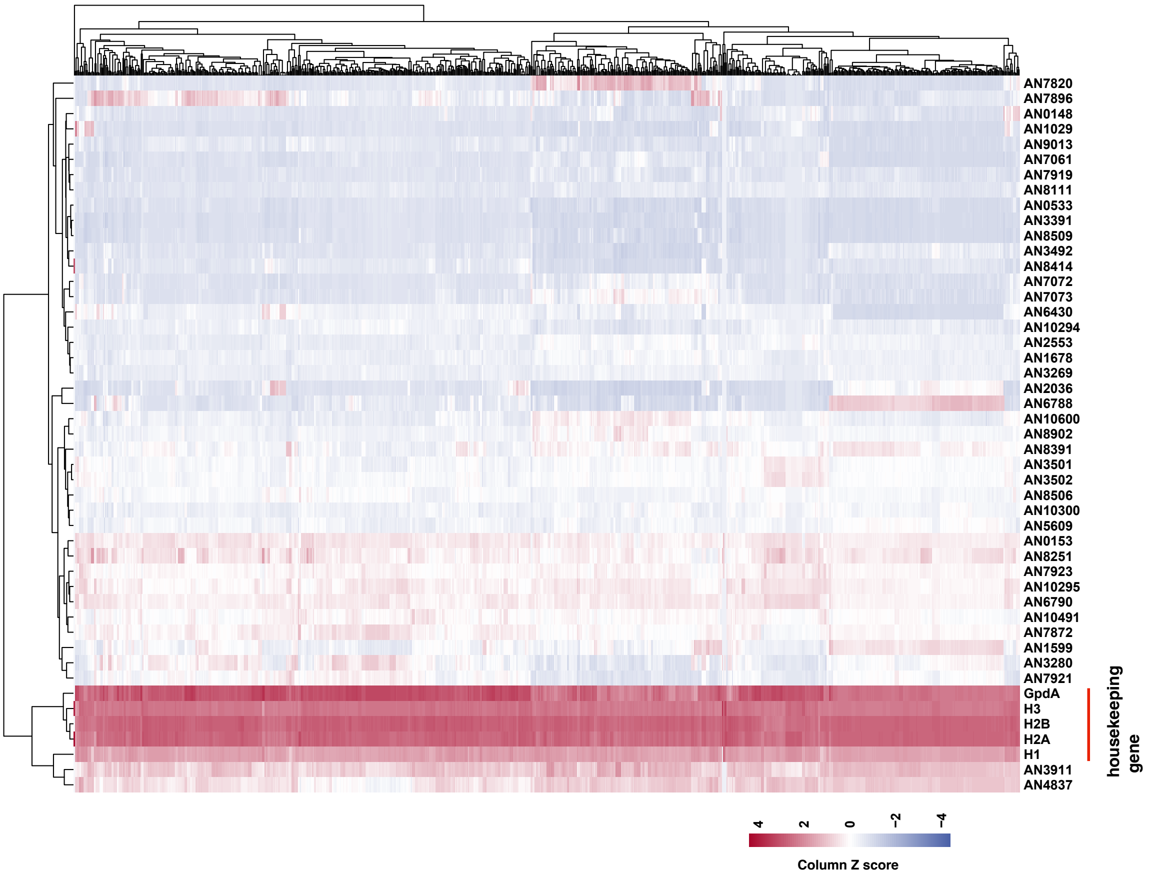

Supplementary Figure 1. A heatmap showing the expression patterns of 5 select housekeeping genes and the chosen SM TFs' genes across different experimental conditions from 878 public RNAseq data.

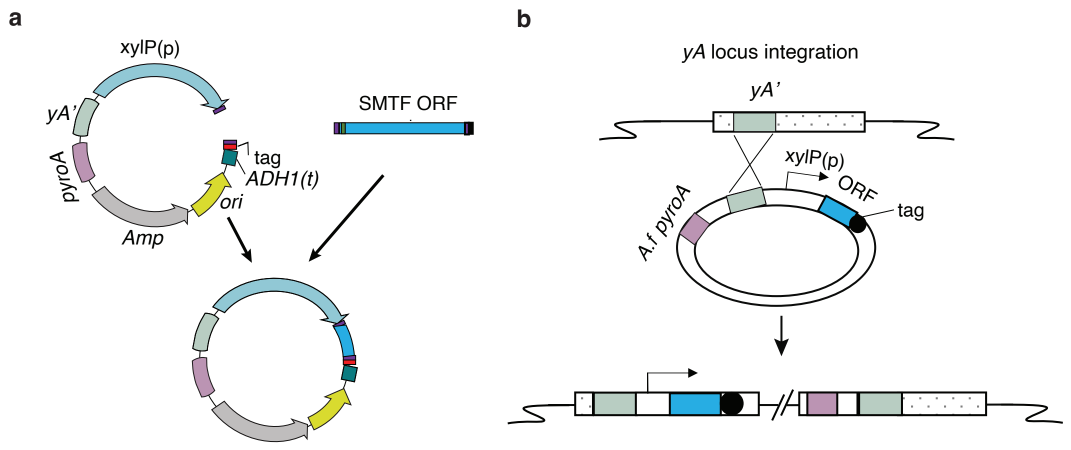

Supplementary Figure 2. Construction and genome integration strategies of TF OE plasmids. a, Schematic diagrams showing the relevant components of the over-expression plasmid and the construction of SM TF over-expression plasmids. **b,** A schematic diagram the integration of the over-expression plasmid in (**a**) at the endogenous *yA* locus and the resulted genomic arrangement.

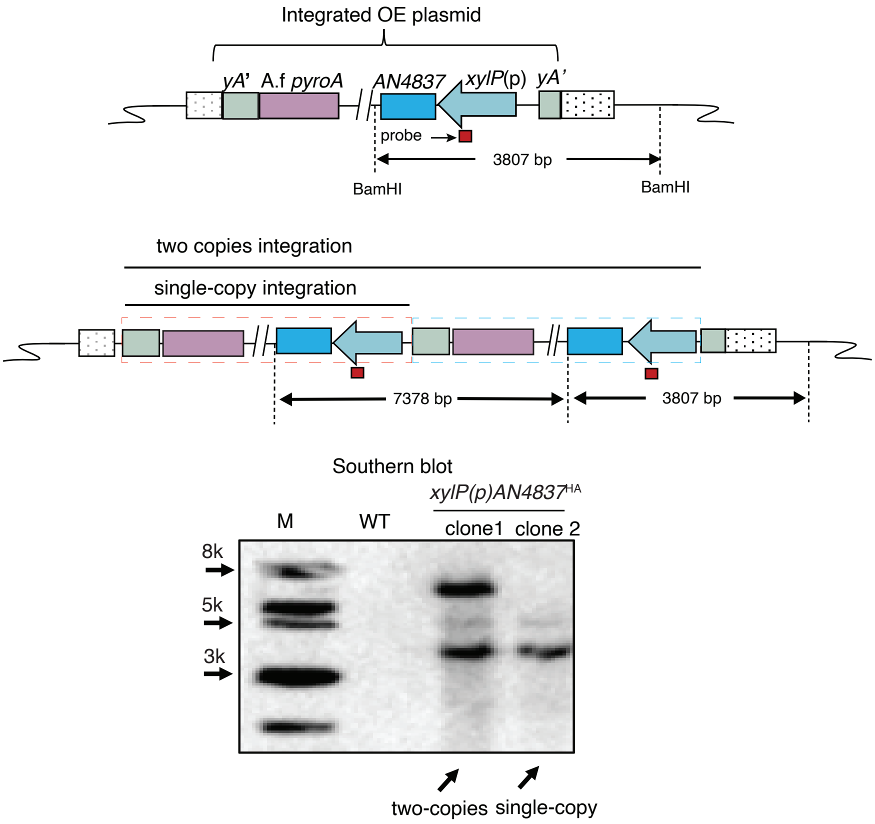

Supplementary Figure 3. The rationale and probe design of Southern Blot analysis for single-copy clone selection-illustrated with AN4837-OE strain. The probe is designed to anneal to a unique site of the plasmid and a common site of different OE plasmids. We located this site within the *xylP* promoter. A restriction enzyme used for genome DNA digestion should have two recognition sites on the OE plasmid and one site in the genome near the plasmid integration site, thereby producing two distinguishable fragments on the agarose gel.

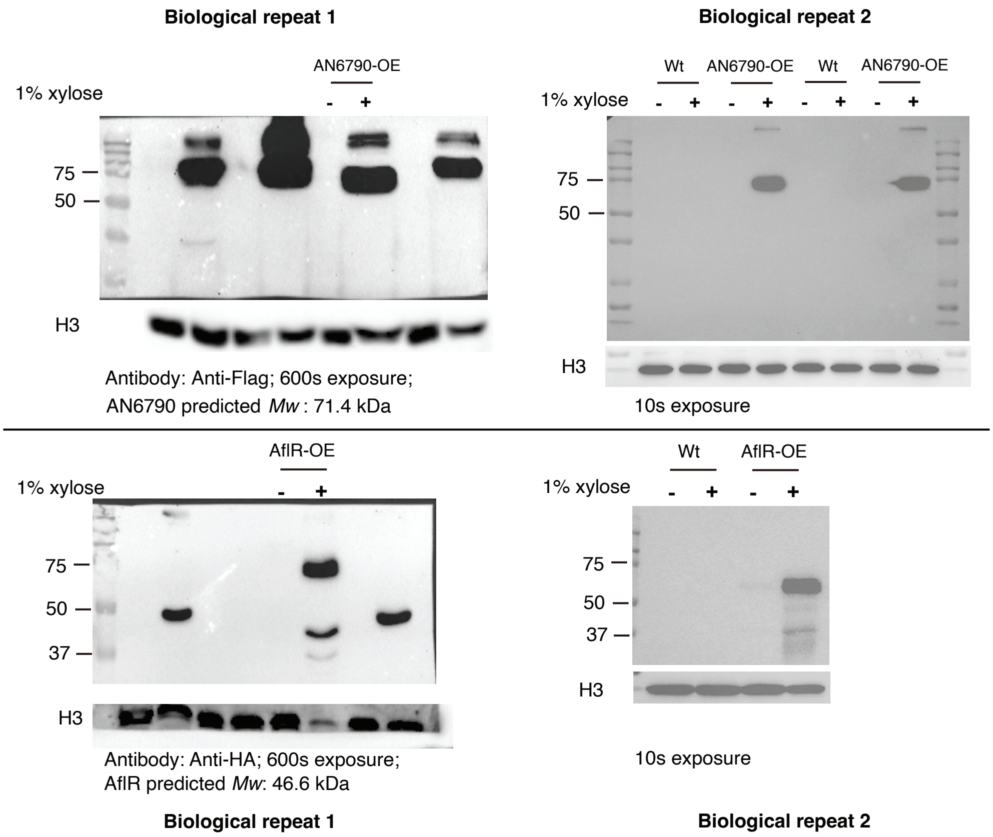

Supplementary Figure 4. Uncropped and unedited Western blot images for Figure 1a. Two biological repeats were performed using protein samples collected on two different days.
