# Supplementary material for: Systematic over-expression of secondary metabolism transcription factors to reveal the pharmaceutical potential of Aspergillus nidulans
Source: Commun Biol. 2025 Oct 9;8:1444. doi: 10.1038/s42003-025-08840-z (PMC12511407; doi:10.1038/s42003-025-08840-z)
Supplement: Supplementary file 3 — Description of Additional Supplementary Materials [file 42003_2025_8840_MOESM3_ESM.pdf]

Description of Additional Supplementary Files

File name: Supplementary Data 1
Description: Source data for Figure 2
